# Net Absorption and Metabolism of β-Hydroxy- β-Methyl Butyrate during Late Gestation in a Pig Model

**DOI:** 10.3390/nu12020561

**Published:** 2020-02-21

**Authors:** Liang Hu, Niels Bastian Kristensen, Uffe Krogh, Peter Kappel Theil

**Affiliations:** 1 Department of Animal Science, Aarhus University, DK-8830 Tjele, Denmarknbk@seges.dk (N.B.K.); uffe.krogh@inrae.fr (U.K.); 2 Institute of Animal Nutrition, Sichuan Agricultural University, No. 211, Huimin Road, Wenjiang District, Chengdu 611130, China; 3 Danish Agriculture & Food Council F.m.b.A. SEGES Agro Food Park 15, DK 8200 Aarhus N, Denmark; 4 PEGASE, INRAE, Agrocampus Ouest, 35590 Saint-Gilles, France

**Keywords:** amino acids, HMB, leucine metabolites, sow, transition

## Abstract

The leucine metabolite, β-hydroxy-β-methyl butyrate (HMB), is widely used in human nutrition and animal production as a nutritional supplement. Although the HMB usage during late gestation has been demonstrated to have a positive effect on fetal development, knowledge on net absorption and metabolism of HMB and impact of HMB on branched chain amino acids (BCAAs) metabolism is lacking. To address this, we conducted a study using pigs during the perinatal period as a model organism. Eight-second parity sows were fitted with indwelling catheters in the femoral artery and in the portal, hepatic, femoral, and mesenteric veins. Eight hourly sets of blood samples were taken starting 30 min before the morning meal on day –10 and day –3 relative to parturition. Four control (CON) sows were fed a standard lactation diet from day –15 and throughout the experiment, and 4 HMB sows were fed the control diet supplemented with 15 mg Ca(HMB)_2_/kg body weight mixed in one third of the morning meal from day –10 until parturition. Blood gases, plasma metabolites, milk compositions, and apparent total tract digestibility of nutrients were measured. Arterial plasma concentrations of HMB (*p* < 0.001), Cys (*p* < 0.001), and Lys (*p* < 0.10) were increased in HMB supplemented sows, while arterial plasma triglycerides concentration was decreased (*p* < 0.05). The net portal recovery of Ala and Asp were increased in HMB sows (*p* < 0.05). Sows fed HMB had increased hepatic vein flow and net hepatic fluxes of Met, Asn, and Gln (*p* < 0.05). In contrast, the femoral extraction rates of Ala and Ser were decreased by dietary HMB supplementation (*p* < 0.05). Dietary HMB treatment and sampling time relative to feeding had an interaction on arterial concentrations, net portal fluxes, and femoral extraction rates of BCAAs. The net portal recovery of HMB was 88%, while 14% of supplemented HMB was excreted through urine and 4% through feces. Moreover, the gastrointestinal tract metabolized 8% while the liver metabolized 12%. Finally, 26% of the daily intake of HMB was secreted via colostrum at the day of farrowing. This study demonstrated that dietary HMB supplementation increased net uptake of amino acids and increased fatty acid oxidation through improving blood flow and insulin sensitivity during the late gestation. Most importantly, oral HMB administration could maintain a stable postprandial absorption and altered metabolism in BCAAs. Net portal flux of HMB at 5.5 to 6.5 h after feeding approached zero, indicating that HMB ideally should be administrated two or three times, daily.

## 1. Introduction

β-hydroxy-β-methyl butyrate (HMB) is a metabolite of the nutritionally essential branched-chain amino acid (BCAA) leucine [1,2]. Human studies have shown that HMB stimulated muscle growth and increased fat oxidation, and therefore, it is commonly used as a dietary supplement for athletes and for bedridden patients to prevent excessive muscle degeneration [3,4]. Moreover, HMB is also widely applied in animal production as a functional additive over the last decades. For late gestating sows, HMB may increase the colostrum fat content or colostrum yield when supplied at 2 to 2.5 g/d during the last 3 to 7 days prior to parturition [1,5]. 

Leucine catabolism normally involves a transamination process to form α-ketoisocaproic acid (KIC), which may be further metabolized into HMB by the enzyme α-ketoisocaproate-dioxygenase [2]. In a study with pigs, it was demonstrated that 2 to 10% of injected leucine was converted to HMB and found in the cytosol [6]. Van Koevering and Nissen also revealed that 34% of injected HMB was excreted in the urine, but otherwise, the fate of HMB remains unknown [6]. Metabolism of BCAAs are known to interact with each other, and it is generally believed that excessive supply of dietary leucine leads to catabolism of isoleucine and valine [7,8]. However, recently excessive supply of isoleucine was shown to reduce growth in post-weaning of pigs [7], whereas, the excessive supply of leucine or valine had no negative effects on growth performance [9,10]. 

Interest in prenatal nutritional programming has increased in both animal and human research. Moreover, it has also been demonstrated in animal studies that prenatal administration of HMB has a positive impact on postnatal growth and development [11,12]. However, there is an insufficient number of studies concerning the comprehensive distribution of metabolism when HMB is orally administered during late gestation. Owing to the high similarity in anatomy and physiology between pigs and humans, the porcine model has been frequently used to study human nutrition and intermediary metabolism [13]. Therefore, the present study was carried out to (1) study the fate of supplemented HMB and dietary amino acids (AA) to reveal how these metabolites are digested, absorbed, metabolized, secreted and excreted and (2) to study whether HMB supplementation interacts with fates of dietary BCAAs. More specifically, the net portal absorption, net portal recovery and net hepatic metabolism of these metabolites were quantified using multi-catheterized sows and measurements of blood flow using a blood flow marker, as described for growing pigs [14]. In addition, venous-arterial differences across the right hind leg were studied to evaluate extraction rates of HMB by muscle tissue. This experiment was carried out with late gestating sows within the scope of another study [15]. 

## 2. Materials and Methods

The present experiment complied with Danish law on the humane care and use of experimental animals (The Danish Ministry of Justice, Animal Testing Act [Consolidation Act No. 1306 of 23 November 2007, as amended by Act No. 612 of June 14, 2011]). The protocols used in the current experiment were reviewed and approved by The Danish Animal Experimentation Inspectorate (Ethic Approval Code: 2008/561-1493).

### 2.1. Animals, Surgical Procedures and Housing

Eight-second parity sows (Danish Landrace × Yorkshire) were used in this study and housed in an intensive care facility at Research Center Foulum, Aarhus University, Denmark. The sows were surgically fitted with indwelling catheters on day 79 ± 3 of gestation, including the right femoral artery, the right femoral vein, the portal vein, the portal hepatic vein and the mesenteric vein as described by Kristensen et al. [14]. After surgery catheters were filled with saline containing heparin (100 IU/mL, Heparin LEO, LEO Pharma A/S, Ballerup, Denmark), benzyl alcohol (0.1%, Benzylalcohol +99%, Sigma-Aldrich, St. Louis, MO), and benzylpenicillin (0.2%, Benzylpenicillin, Panpharma, NordMedica A/S, Copenhagen, Denmark). After surgery, the sows were individually housed in pens (2.0 × 2.7 m) with the partly slatted floor (3.0 m^2^) until end of lactation. Sows were fitted in farrowing railings from d –11 and onwards. Room temperature was kept at 20 °C, and the light was turned on from 0700 to 1830 h and again during the evening meal (2400 to 0030 h), except when the sows were farrowing (light turned on 24 h per d). At the end of the experiment (day 28 of lactation), the positions of the catheters were investigated by autopsy to ensure correct placement.

### 2.2. Diets and Feeding

Sows were allocated by body weight (BW) into two groups (4 sows/group). The control sows received a control (CON) diet (Supplementary Table S1) from day –15 and throughout the experiment, and HMB sows were fed the CON diet supplemented with 15 mg Ca(HMB)_2_/kg individual BW mixed in the morning meal from day –10 prior to expected farrowing until parturition, then fed the control diet until day 28. The Ca(HMB)_2_ was purchased from VWR-Bie and Berntsen (Herlev, Denmark). Feed allowance was in accordance with the recommendations from the Danish Pig Research Center (VSP, Copenhagen, Denmark). Chromic oxide (0.2%) was added to the diet as a digestibility marker for measuring the apparent total tract digestibility (ATTD) of nutrients. Feed was supplied three times daily in equal portions at 0800, 1600, and 2400 h, and the feed intake was registered and feed leftovers were recorded and removed daily between 0800 and 1600, except on sampling days, where leftovers from the night meal were removed before the first sampling and leftovers from the morning meal were removed 30 min after feeding to ensure accurate recording of feed intake directly related to nutrient absorption and metabolism. Feed intake corrected for refusals were recorded on a daily basis. The piglets had no access to creep feed, but both sows and piglets were offered water ad libitum.

### 2.3. Sampling and Data Collection

Blood sampling was taken on d –10 and d –3 relative to expected parturition. On sampling days, continuous infusion of the blood marker, *para*-amino hippuric acid (*p*AH), into the mesenteric vein was initiated 1 h before the first blood sample to obtain a steady state of plasma *p*AH. The *p*AH infusate contained 175 mmol/L of *p*AH, was adjusted to pH 7.4, sterile filtered (0.22 µm, FPE 214-500, JET Bio-Filtration Products Co., Ltd., Guangzhou, China), and autoclaved. The infusion rate of *p*AH was 79.4 ± 9.3 mL/hour. Eight sets of blood samples were simultaneously drawn from four catheters (the artery, the portal vein, the hepatic vein and the femoral vein) at hourly intervals from 0.5 h before to 6.5 h after feeding. Whole blood was collected for blood gas measurements, while plasma was obtained by centrifuging the blood sample at 1558 × *g* at 4 °C for 12 min and stored at −20 °C until analysis.

The sows were weighed on day –10, day 2, and day 28 relative to parturition. All piglets were weighed at birth, and weighed again 24 h after the birth of the first piglet (BFP) for determination of colostrum yield [16]. Individual piglet weights were registered on day 2 and day 28 after parturition. The piglets did not receive any feed, but had free access to water at all times. At 24 h after BFP, the litters were equalized to 12 piglets within the experimental groups. Colostrum samples were taken immediately and at 24 h after birth, and milk was collected after blood sampling on d 3 and 17, after a 0.3 mL i.v. injection of oxytocin. Spontaneous urine and feces samples were collected on blood sampling days, and aliquots were stored at −20 °C for later analysis. 

### 2.4. Analytical Procedures

*Diets*. Dry matter (DM) content was measured by drying to constant weight (approximately 20 h) at 103 °C, and ash was analyzed according to the AOAC (2000) method no 942.05. Feed gross energy (GE) was determined using an adiabatic bomb calorimeter (Parr Instrument Company, Moline, Illinois, USA). The crude fiber was analyzed according to the AOAC method (method 978.10, AOAC, 2007). Nitrogen content was measured by the Kjeldahl method (Kjeltec 2400, Foss, Hillerød, Denmark) according to the manufactures instructions and protein was calculated as Nitrogen × 6.25. Fat was extracted with diethyl ether after HCl hydrolysis according to the Stoldt procedure [17]. Amino acids were quantified after hydrolysis for 23 h at 110 °C with or without performic acid oxidation using ion exchange chromatography and photometric detection after ninhydrin reaction. Chromic oxide in feed was measured spectrophotometrically after oxidation to chromate.

*Whole blood*. Hematocrit was immediately determined in arterial blood samples by centrifugation in capillary tubes at 13,000 × *g* for 6 min at ambient temperature. Blood pH and blood gasses were measured using 2 ABL700 Blood Gas Analyzers (Radiometer, Copenhagen, Denmark). 

*Plasma*. For determination of *p*AH concentration, plasma was deacetylated before analysis by the method as described by Harvey et al. [18], and determined using a continuous flow analyzer (Seal Analytical Ltd., Burgess Hill, UK). The concentrations of urea were determined by the method described by Marsh et al. [19]. The HMB concentration was analyzed, after ethyl chloroformat derivatization, determined using gas chromatography/mass spectrometry (GC-MS; Finnigan trace GC ultra, CTC analytics). Plasma AA concentration was determined by GC-MS following ethyl chlorformat derivatization. Glucose and lactate were determined using D-glucose oxidase and L-lactate oxidase, respectively (YSI 7100, YSI inc., Yellowstone Springs, OH). Insulin concentration was analyzed using a solid phase, two-site flouroimmunometric assay (AutoDELFIA, Wallac Oy, Turku, Finland). Plasma concentration of volatile fatty acids (VFA) was determined by GC-MS following 2 Chloro-Ethyl-Chloroformat derivatization [20]. Non-esterified fatty acid (NEFA) concentration in plasma was analyzed using commercial kits for Cobas Mira autoanalyzer (Triolab A/S, Brøndby, Denmark).

*Urine and feces*. Urinary HMB was analyzed as described for plasma. Feces content of nitrogen, DM, ash, chromium oxide, energy, crude fiber, and fat were determined as for the feed. Feces content of HMB was determined using GC-MS.

*Colostrum and milk*. Milk composition was analyzed for fat, lactose, protein, and DM concentrations by infrared spectroscopy (Milkoscan 4000; Foss MilkoScan, Hillerød, Denmark). The estimated values obtained by the Milkoscan method were corrected using linear models based on reference methods [21]. Concentrations of total AA in freeze-dried milk samples were analyzed using a Biochrom 30+ AA analyzer (Biochrom, Cambridge, UK) by ion exchange chromatography according to the European Union after hydrolysis in 6 M HCl for 23 h. Sulfur containing AA was separately liberated by oxidation in a hydrogen peroxide and performic acid solution for 16 h prior to hydrolysis. Milk HMB content was analyzed as described for plasma.

### 2.5. Calculations and Statistical Analysis

Milk intake of the piglets on d 3 and 17 DIM was calculated according to Theil et al. [22]. Sow milk yield was calculated as the sum of milk intake of the piglets within each litter and DIM. Energy and nutrients ATTD were calculated as described by Stein et al. [23]. Plasma flows and blood flows in the portal vein, hepatic vein and the hepatic artery as well as net portal flux (NPF), net hepatic flux (NHF), net portal recovery, and hepatic extraction were calculated according to Hu et al. [24]. HMB intake (g/d) = sow BW × 0.015 × 0.808, where 0.015 corresponds with 15 mg/kg BW and 0.808 accounts for HBM content in Ca(HMB)_2_ ⋅ 1 H_2_O (molar mass = 292.35 g/mol), and when converting into mmol/L or mmol/h, 2 mol of HMB for each mole of Ca(HMB)_2_ was taken into account. HMB excretion in urine was calculated from equations below (Equations (1) and (2)), where urine production was calculated according to Feyera et al. [25] as the infusion rate of *p*AH divided by urinary *p*AH concentration:

HMB excretion in urine (g/d) = [HMB concentration in HMB sow’s urine (mmol/mL) × urine volume (mL/d) − HMB concentration in CON sow’s urine (mmol/mL) × urine volume (mL/d)] × 118.13 (g/mol) × 0.001 (mol/mmol)(1)

HMB excretion in urine relative to intake (%) = HMB excretion in urine (g/d)/ HMB intake (g/d) × 100(2)

HMB content in feces was calculated according to the following equations (Equations (3)–(5)):

Undigested DM (kg/d) = Average daily feed intake (kg/d) × [1 − DM digestibility (%)](3)

HMB content in feces (g/d) = Undigested DM (kg/d) × HMB concentration in feces (mmol/g) × 118.13 (g/mol) × 0.001 (mol/mmol)(4)

HMB in feces relative to intake (%) = HMB content in feces (g/d)/HMB intake (g/d) × 100(5)

HMB metabolized by gastrointestinal tract (GIT) was calculated according to the following equation (Equation (6)):

HMB metabolized by GIT relative to intake (%) = 100 − HMB in feces relative to intake (%) − Net portal recovery of HMB (% of intake)(6)

HMB production in colostrum/milk was calculated according to the following equation (Equations (7) and (8)):

HMB secretion through colostrum/milk (g/d) = yield (kg/d) × HMB concentration in colostrum/milk (mmol/L) × 118.13 (g/mol) × 1 (kg/L) × 0.001 (mol/mmol)(7)

HMB secretion through colostrum/milk relative to intake (%) = HMB secretion through colostrum/milk (g/d)/HMB intake (g/d) × 100(8)

HMB metabolism by the liver was calculated according to the following equation (Equations (9) and (10)):

HMB metabolism by liver (g/d) = Net hepatic flux of HMB (mmol/h) × 24 h/d × 118.13 (g/mol) × 0.001 (mol/mmol)(9)

HMB metabolism by liver relative to intake (%) = HMB metabolism by liver (g/d)/ HMB intake (g/d) × 100(10)

The MIXED procedure of SAS (SAS Inst. Inc., Cary, NC) was applied in the statistical analysis using a model, including the fixed effects of treatment (CON, HMB), DIM (–10, –3), time relative to feeding (−0.5, 0.5, 1.5, 2.5, 3.5, 4.5, 5.5, 6.5), and all 2-way interactions between fixed effects. The sow was included as a random effect using a compound symmetry covariance structure for accounting for repeated measurements within sows across DIM. To account for repeated measurements within a sampling day, a first order autoregressive covariance structure was applied using a repeated statement. Data were presented as means ± SEM. A probability value of *p* < 0.05 was considered as statistically significant and *p* < 0.10 was considered as a tendency.

## 3. Results

### 3.1. Feed intake, Sow Performance and Digestibility

According to the experimental design, HMB sows ingested on average 3.2 g/d of HMB, whereas, CON sows did not ingest any HMB from d –10 up until farrowing (*p* < 0.001, Table 1). Feed intake, sow BW change, ATTD, and litter performance were not affected by HMB supplementation (*p* > 0.05). 

### 3.2. Yield and Composition of Colostrum and Milk

**Figure 1:**
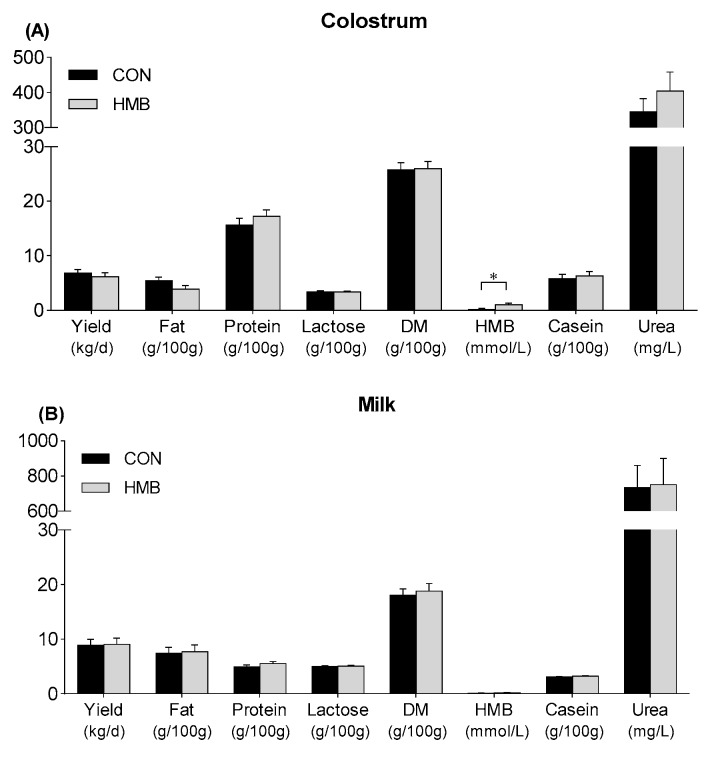
Milk yield and milk composition of colostrum (**A**) and milk (**B**) in control (CON) and HMB-supplemented (HMB) sows. Data are expressed as the mean ± SEM. Sows were regarded as the experimental units, *n* = 4 for each group. The differences between groups were indicated by an asterisk (*p* < 0.05). CON = control; HMB = β-hydroxy-β-methyl butyrate; DM = dry matter.

The yield and composition in both colostrum and milk were not affected by HMB supplementation (*p* > 0.05, Figure 1), except that HMB concentration of colostrum was higher for HMB sows than for CON sows (*p* = 0.04, Figure 1). Moreover, HMB supplementation increased the milk concentrations of Met (*p* = 0.047) and tended to increase the milk concentrations of Glu (*p* = 0.07, Table 2). All milk amino acid concentrations were affected by DIM (*p* < 0.01), but no interaction between treatment and DIM was observed (*p* > 0.05).

### 3.3. Arterial Variables

**Figure 2:**
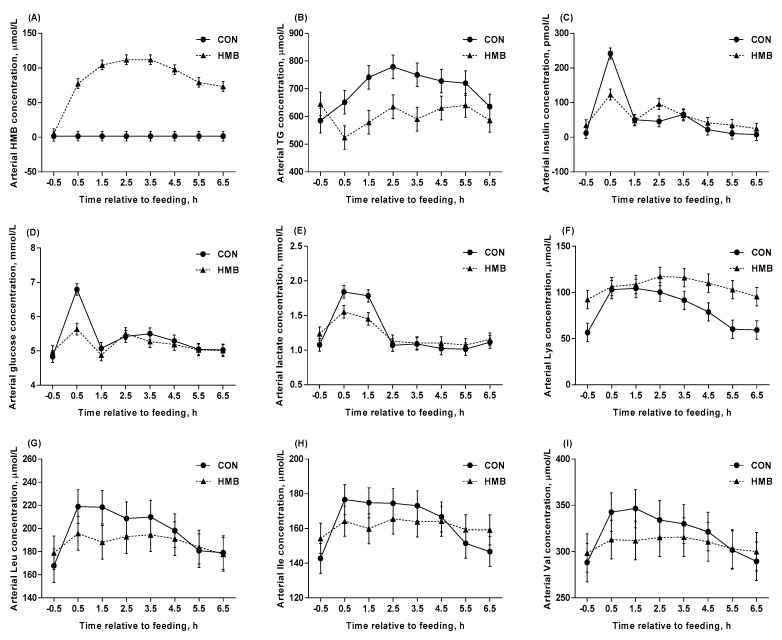
Arterial concentrations of HMB (**A**), TG (**B**), insulin (**C**), glucose (**D**), lactate (**E**), Lys (**F**), Leu (**G**), Ile (**H**), and Val (**I**) in control (CON) and HMB-supplemented (HMB) sows during late gestation. Data are expressed as the mean ± SEM. Sows were regarded as the experimental units, *n* = 4 for each group. Interaction of treatment and sampling time after feeding were observed for arterial concentration of HMB (*p* < 0.001), TG (*p* = 0.06), insulin (*p* < 0.001), glucose (*p* < 0.001), lactate (*p* = 0.002), Lys (*p* = 0.003), Leu (*p* = 0.02), Ile (*p* = 0.006), and Val (*p* = 0.006), respectively. CON = control; HMB = β-hydroxy-β-methyl butyrate; TG = triglycerides.

Sows fed HMB had increased arterial plasma concentrations of HMB (*p* < 0.001, Table 3) and Cys (*p* < 0.001), and had a tendency to increase Lys concentration (*p* = 0.09) compared to CON sows, whereas, HMB sows had lower arterial plasma concentration of TG (*p* = 0.046) compared to CON sows. The arterial concentrations of CO_2_, insulin, Leu, Val, Asn, Tyr, Gln, acetate and propionate at DIM –3 were lower than that at DIM –10 (*p* < 0.05). Interaction between treatment and DIM was observed for arterial hematocrit (*p* = 0.004), O_2_ (*p* = 0.005), Ile (*p* = 0.03), Leu (*p* = 0.01), Asn (*p* = 0.02), and Ser (*p* = 0.02). All measured arterial concentrations of metabolites, except propionate and butyrate, were affected by ST (*p* < 0.05). Interactions between treatment and ST were observed for arterial HMB (*p* < 0.001), TG (*p* < 0.10), insulin (*p* < 0.001), glucose (*p* < 0.001), lactate (*p* = 0.002), and Lys (*p* = 0.003) are illustrated in Figure 2. Interactions between treatment and ST for arterial hematocrit (*p* = 0.04), Thr (*p* = 0.008), Trp (*p* = 0.009), Ile (*p* = 0.006), Leu (*p* = 0.02), Val (*p* = 0.006), Phe (*p* = 0.003), Ala (*p* < 0.001), Asn (*p* < 0.001), Asp (*p* = 0.02), Ser (*p* = 0.003), Tyr (*p* = 0.009), Gln (*p* = 0.04), and butyrate (*p* = 0.05) are shown in Table 3. 

### 3.4. Blood Flow, Net Portal Flux, and Net Portal Recovery

**Figure 3:**
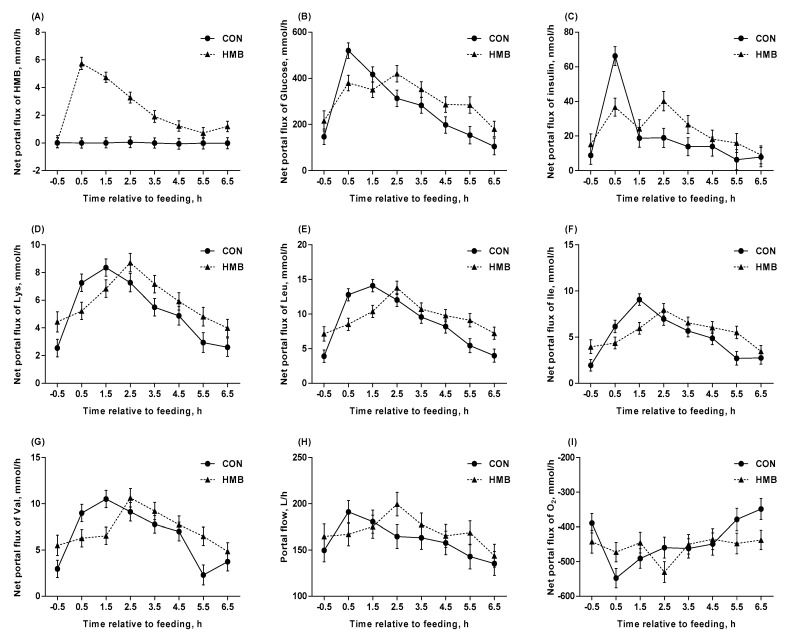
Net portal fluxes of HMB (**A**), glucose (**B**), insulin (**C**), Lys (**D**), Leu (**E**), Ile (**F**), Val (**G**), portal flow (**H**), and net portal flux of O_2_ (**I**) in control (CON) and HMB-supplemented (HMB) sows during late gestation. Data are expressed as the mean ± SEM. Sows were regarded as the experimental units, *n* = 4 for each group. Interaction of treatment and sampling time after feeding were observed for net portal fluxes of HMB (*p* < 0.001), glucose (*p* < 0.001), insulin (*p* < 0.001), Lys (*p* = 0.004), Leu (*p* < 0.001), Ile (*p* < 0.001), Val (*p* = 0.001), portal flow (*p* = 0.03), and O_2_ (*p* = 0.04), respectively. CON = control; HMB = β-hydroxy-β-methyl butyrate.

Dietary supplementation of HMB increased the net portal fluxes of HMB (*p* < 0.001, Table 4) and Cys (*p* = 0.005), and tended to increase the net portal flux of NEFA (*p* = 0.09), while it tended to decrease the net portal flux of acetate (*p* = 0.09). The net portal fluxes of insulin, lactate, and Ala at DIM –3 were lower than that at DIM –10 (*p* < 0.05). Interaction between treatment and DIM was observed for portal flow, net portal fluxes of insulin, Met and Asn (*p* < 0.05). All measured net portal fluxes, except for urea, NEFA, TG and VFA, were affected by ST (*p* < 0.05). Interactions between treatment and ST was observed for HMB, glucose, insulin, Lys, Leu, Ile, Val, portal flow, and net portal flux of O_2_ are illustrated in Figure 3. Interaction between treatment and ST was observed in most measured net portal fluxes (*p* < 0.05, Table 4 and Figure 3), except for CO_2_, lactate, urea, NEFA, TG, Asp, Glu, Gly, Gln and VFA. Relative to feeding, the net portal fluxes of most AA peaked 2.5 h after feeding in sows fed with HMB, while they peaked 1.5 h after feeding in CON sows (Figure 3). Dietary supplementation of HMB increased the net portal recovery of Ala (*p* = 0.04, Table 5) and Asp (*p* = 0.003). All net portal recovery of AA did not differ across DIM (*p* > 0.05). The net portal recovery of HMB in sows fed HMB was 88%.

### 3.5. Net Hepatic Flux

Sows fed HMB increased the hepatic vein plasma flow (*p* = 0.002, Table 6), net hepatic fluxes of Met (*p* < 0.001), Asn (*p* = 0.04), and Gln (*p* = 0.04), and tended to increase the hepatic artery plasma flow (*p* = 0.06), net hepatic fluxes of Phe (*p* = 0.06), and Gly (*p* = 0.07). Net hepatic fluxes of HMB, urea, TG, Trp, Ile and Val were more negative at DIM –3 than at DIM –10 (*p* < 0.05). Interaction between treatment and DIM was observed for net hepatic fluxes of HMB (*p* = 0.02) and Met (*p* = 0.02). Net hepatic fluxes of insulin, lactate, urea, Lys, Met, Ile, Leu, Val, Phe, Ala, Asn, Asp, Cys, Glu, Ser and Tyr were affected by ST (*p* < 0.05). Interaction between treatment and ST was observed for net hepatic fluxes of insulin (*p* < 0.001), Thr (*p* = 0.04), Ala (*p* = 0.03), Asp (*p* = 0.01) and Ser (*p* = 0.002). 

### 3.6. Femoral Extraction

**Figure 4:**
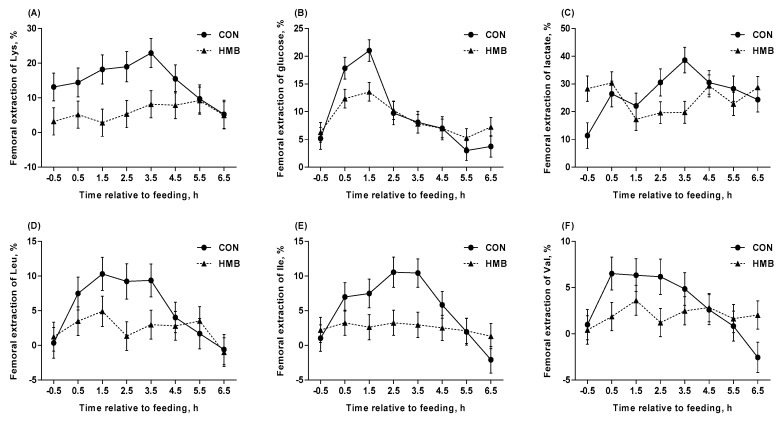
Femoral extractions of Lys (**A**), glucose (**B**), lactate (**C**), Leu (**D**), Ile (**E**), and Val (**F**) in control (CON) and HMB-supplemented (HMB) sows during late gestation. Data are expressed as the mean ± SEM. Sows were regarded as the experimental units, n = 4 for each group. Interactions of treatment (CON, HMB) and sampling time after feeding were observed for femoral extractions of Lys (*p* = 0.001), glucose (*p* = 0.004), lactate (*p* = 0.006), Leu (*p* = 0.10), Ile (*p* = 0.01), and Val (*p* = 0.04), respectively. CON = control; HMB = β-hydroxy-β-methyl butyrate.

Dietary supplementation of HMB decreased the femoral extractions of Ala and Ser (*p* < 0.05; Table 7), and tended to decrease the femoral extractions of insulin (*p* = 0.09) and Glu (*p* = 0.09). Femoral extractions of glucose, Lys, His, Glu, and Gln at DIM –3 were lower than that at DIM –10 (*p* < 0.05). Interaction between treatment and DIM was observed for the femoral extraction of propionate (*p* < 0.05). Most of femoral extractions were affected by ST (*p* < 0.05), except for HMB, urea, NEFA, TG, His, Glu, Gly, Gln, acetate, butyrate and isovalerate. Interaction between treatment and ST was observed for femoral extractions of glucose (*p* = 0.004), lactate (*p* = 0.006), Lys (*p* = 0.001), Ile (*p* = 0.01), Val (*p* = 0.04), Phe (*p* = 0.01), Ala (*p* = 0.02), Cys (*p* = 0.02), and propionate (*p* < 0.001; Table 7 and Figure 4).

### 3.7. HMB Metabolism

**Figure 5:**
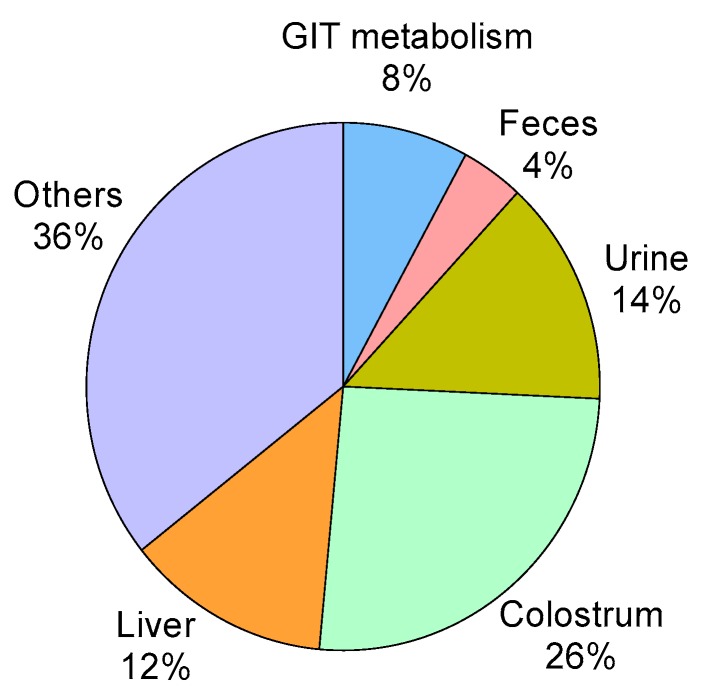
β-hydroxy-β-methyl butyrate (HMB) distribution in sow tissues and metabolites (liver, gastrointestinal tract, colostrum, urine, feces, and others) refer to dietary HMB intake. GIT, gastrointestinal tract. The amount of HMB secreted into colostrum is calculated under the assumption that it is taken up by the mammary gland at the day of farrowing [26]. At day –10 and –3, the 26% representing colostrum most likely contributes to “Others”, as this category is calculated as a difference.

The partitioning of HMB in the HMB supplemented sows revealed that ATTD of HMB was 96% and 4% was lost through feces (Figure 5). Furthermore, 8% of HMB disappeared in the GIT, as evaluated by the difference between the net portal recovery rate (88%) and ATTD. The hepatic uptake amounted to 12% of the HMB intake, whereas, HMB secreted via colostrum and excreted through urine amounted to 26% and 14%, respectively, relative to intake. Finally, 36% of HMB relative to intake could not be accounted for quantitatively, but include uptake to muscles, as demonstrated for the hindleg (referred to as “others” in Figure 5).

## 4. Discussion

### 4.1. Effects of HMB Supplementation on Reproductive Performance in Late Gestation

Over the last decades, there has been increased interest in HMB for both animal and human research, due to its nutritional role [27]. The addition of HMB in human has been associated with beneficial health effects for people suffering from several diseases [28]. In animals, numerous studies have shown that dietary supplementation with HMB improved growth and production performance [29]. In the current study, maternal HMB supplementation during the late gestation had no influence on birth weight of piglets, which is in agreement with previous studies [1,5], although other studies have reported increased birth weight in response to maternal HMB supplementation [12,30]. These contradictory results may be due to the varying lengths of HMB administration, meal frequency, the doses of HMB that have been given and not least the number of animals included in the different studies. In a former study, sows fed with HMB at 2 g/d in three equal daily meals for three days prior to parturition increased the fat concentration of sow’s colostrum and in turn improved litter growth performance [1]. Flummer and Theil demonstrated that HMB supplemented to sows improved the colostrum production, but inhibited piglet growth at peak lactation [5], which could be due to compromised milk production at peak lactation, due to excessive depletion of labile fat or protein depots around parturition. In the present study, dietary supplementation of HMB increased some AA concentrations (Met, Glu and Pro) in milk, but had no effect on litter performance. This was supported by the milk yield, which did not differ between CON and HMB sows. 

### 4.2. Effects of HMB Supplementation on Blood Flow and O_2_ Consumption

The portal blood flow may reflect a circulatory response to gastrointestinal function related to the process of digestion and absorption [31]. In the current study, dietary supplementation of HMB increased the portal flow and net portal flux of O_2_ across sampling time after feeding and suggested that HMB supplementation is beneficial for enhancing transport of absorbed nutrients, and this is supported by improved net portal recovery of AA (some were statistically different, some were numerically greater). Also, dietary supplementation of HMB increased the hematocrit concentration of arterial blood across sampling time after feeding. It is well known that an increased hemoglobin content linked to an increased hematocrit would increase the efficiency of O_2_ transport [32]. In support of this, HMB supplementation in endurance-trained cyclists has positive effects on aerobic performance by delaying the onset of blood lactate accumulation and extending the time until maximal O_2_ consumption was reached [33]. 

The liver plays an essential role in intermediary metabolism and account for a substantial proportion of the whole animal O_2_ consumption [34]. In this study, dietary supplementation of HMB clearly increased the plasma flow in hepatic vein (197 to 260 L/h, *p* = 0.002), which was driven by the increase in hepatic artery plasma flow (38 to 90 L/h, *p* = 0.06), while portal plasma flow was unaffected. The increased hepatic plasma flow resulted in a numerically increased net hepatic uptake of O_2_. Net hepatic O_2_ flux were −487 and −617 mmol/h (*p* = 0.19) for CON and HMB sows, respectively. A negative relationship between blood pressure and plasma flow has been proven in human researches [35,36]. Moreover, Nissen et al. have reported that a decrease in systolic blood pressure was observed in humans who were fed 3 g HMB/d [37]. This change in blood pressure may be attributed to the additional calcium intake, due to the HMB supplementation in the form of a calcium salt, and there is experimental evidence showing that calcium supplementation can affect blood pressure [38,39]. Taken together, the increased plasma flow of hepatic vein and in hepatic artery might be ascribed to the decreased blood pressure of sows supplemented with Ca(HMB)_2_.

### 4.3. Effects of HMB Supplementation on Amino Acids Metabolism

In the current study, dietary supplementation of HMB in sows during late gestation was associated with the increased arterial concentration of Lys and Cys. Results obtained in our study are in accordance with the previous investigations on newborn pigs subjected to maternal oral administration of 0.05 g/kg of Ca(HMB)_2_, which reported that plasma concentrations of Glu, Gly, Val, and Tyr increased by 32%, 30%, 23% and 52%, respectively [30]. Moreover, available data from studies on growing turkeys have shown that 15-week administration with HMB increased concentration of Cys, Glu, Val, Asp, Glu, Pro, Ala, Ile, Leu and Phe, whilst body weights did not differ in comparison to control birds [40]. In studies on pigs subjected to long-term fundectomy (the fundus and glandular part of the cardiac region of the stomach were resected), HMB improved plasma concentration of Met, Thr, Val, Leu, Tyr, Try and Arg, depending on blood sampling time, since last oral dosage of HMB [41]. Taken together, these results indicate an anabolic property of HMB on AA metabolism.

The small intestine of pigs is crucial for the metabolism of AA and nitrogen recycling [42]. Extensive investigations over the past two decades have indicated extensive catabolism of AA by the portal-drained viscera of pigs and humans [32]. The net portal recovery of essential AA are usually stable on 50–70% after catabolizing by the small intestine in first-pass metabolism [43], which is accordant with our results. Dietary HMB supplementation significantly increased the net portal recovery of some AA (Lys, Ala, Asp and Ser), suggesting that the rate of metabolism of these AA by gastrointestinal tissues (as oxidative fuel) were decreased. In addition, the interaction between treatment and sampling time revealed a more uniform net portal uptake of AA after feeding, and hence, lower maximum absorption peaks for HMB sows as compared with CON sows for all AA except Asp, Glu, Gly, and Gln. These results suggest that HMB supplementation preferably should be done at every single meal to achieve a more constant net absorption rate of nutrients. 

The liver plays an important role in the intermediary metabolism of AA; however, there are few reports in the literature describing the net AA metabolism by the liver in nonruminants, particularly in the fed state [44]. In the current study, the hepatic extraction of AA (Met, Thr, Phe, Ala, Asn, Glu, Gly, and Tyr) in HMB supplemented sows were lower than CON sows (data not shown). The decreased hepatic extractions of these AA indicate more AA will be release to peripheral tissues, due to a lower fractional hepatic uptake in the liver. In support of this, we found that the milk AA compositions (Met, Glu and Pro) in HMB supplemented sows were higher than CON sows. Branched chain AA, as the most abundant of essential AA, are not only the substrates for the synthesis of nitrogenous compounds, but they also serve as signaling molecules regulating the metabolism of glucose, lipid, and protein synthesis [45]. It is well known that HMB is responsible partly for the protein anabolic effect of Leu, while neither Ile nor Val have this unique feature, but typically interactions between BCAAs are observed [46]. In the current study, dietary HMB supplementation and sampling time had an interaction on the arterial concentrations, net portal fluxes, and femoral extractions of BCAAs. These results suggested that orally administered HMB could maintain a more stable absorption and metabolism of BCAAs through monitoring arterial concentrations and femoral extractions of BCAAs and delaying the peak of net portal fluxes of BCAAs. In contrast, no impact of HMB or treatment × sampling time interaction was found for BCAAs metabolism in the liver, and support that BCAAs are mainly oxidized peripherally [47]. 

Numerous studies have suggested HMB is responsible for inhibiting proteolysis and for modulating protein turnover in vitro and in vivo. Administration with HMB in humans undergoing exercise resulted in increased muscle mass accretion that was associated with inhibition of muscle proteolysis [41]. In the current study, the femoral extractions of Ala, Glu and Ser were markedly decreased by HMB supplementation, which indicated that HMB supplementation could inhibit muscle proteolysis. Similar findings were reported by Nissen et al. who demonstrated that ingestion of 3.0 g dose of Ca(HMB)_2_/d resulted in decreased release of creatine kinase, an indicator of muscle damage, and 3-methylhistidine, an indicator of muscle protein breakdown [48].

### 4.4. Metabolic Fate of HMB

In the current study, HMB was net absorbed and in large quantities shortly after feeding, indicating that this occurs either in the stomach or in the small intestine, because HMB concentration in plasma peaked around 30 min after ingestion. However, the peak of HMB concentration differs from other studies. There may be a number of factors that influence HMB levels in the plasma. In a human study, Vukovich et al. found that 1 g of HMB-Ca resulted in a peak HMB level in plasma two hours following ingestion, while 3 g resulted in peak HMB levels 60 min after ingestion at 300% greater plasma concentrations (487 vs. 120 nmol/ml) [49]. Moreover, comparison of 0.8 g of HMB-FA (HMB in free acid form) to 1.0 g HMB-Ca (equivalent amounts of HMB) resulted in a doubling of peak plasma levels already after 30 min in the HMB-FA compared with 120 min after HMB-Ca consumption [50]. Another study showed that the peak of HMB concentrations was delayed by an hour (from 60 to 120 min) when the HMB-Ca dosage was combined with 75 g of glucose [49]. Taken together, the peak time of appearance of HMB in plasma following ingestion is dependent on the dose, the source of HMB (HMB-FA or HMB-Ca), and whether or not it is consumed with additional nutrients. These findings suggested that microbial fermentation in the stomach or metabolism in the epithelium of the stomach or small intestine is influenced by HMB.

Numerous studies have reported that the effects of oral administration of HMB on health status and growth performance both in human and animals, but the metabolic fate of HMB is not fully understood. In this study, HMB metabolism was studied quantitatively to understand the metabolic fate of HMB by using multiple catheters and an external blood flow marker in a sow model during late gestation. As a result, approximated 88% (net portal recovery) of HMB intake was net absorbed to the portal vein, and only 4% of the remaining was lost through feces (96% ATTD). The gastrointestinal tract, as the main absorption site, can catabolize HMB by the intestinal mucosa during the first pass, as reflected by 8% of HMB was removed by the GIT in this study. The primary metabolic fate of HMB is its conversion to β-hydroxy-β-methylglutaryl-coenzyme A, which is a committed step for cholesterol synthesis, but it is unclear what percentage of cholesterol is derived from HMB [2]. In the current study, approximately 12% of HMB was catabolized in the liver, and approximately 26% of HMB was released into colostrum if it is assumed that HMB transfer to colostrum occurs after parturition starts [26]. Another fate of HMB loss was excretion in the urine, which accounted for 14%. This finding indicates that the kidney does not actively reabsorb HMB. In a former study, approximately 34% of HMB was lost this way in pigs and sheep [6]. However, a clinical study has shown that only 15% of a 1 g dosage of calcium HMB was excreted in the urine, which is consistent with our results [50]. In addition, approximately 36% of HMB in this study could not be accounted for, because HMB uptake to muscles cannot be measured quantitatively by the multi-catheterized technique. However, the extraction rate of HMB by the femoral muscle support that muscles do indeed metabolize HMB. Metabolism of HMB in skeletal muscle may constitute a greater proportion in late gestation, where HMB is not secreted in colostrum.

### 4.5. Effects of HMB Supplementation on Glucogenic, Ketogenic Substrates and Insulin Secretion 

The role of HMB in glucose and insulin homeostasis is controversial [3,51]. Few reports have emerged showing glucose intolerance and elevation in plasma insulin level after HMB administration. In the current study, we found that dietary HMB supplementation decreased the arterial concentrations and net portal fluxes of glucose and insulin after 30 min of feeding, which suggests that HMB administration improve the insulin sensitivity to a certain extent. In support of this, Sharawy and coworkers reported that dietary supplementation with HMB might attenuate insulin resistance induced by a high fructose diet in rats through inhibition of glucose transporter-2 in the liver [52]. Recent studies have revealed that HMB supplementation may alter energy metabolism, as evidenced by increased fat loss during exercise [53]. In fact, HMB has been claimed to stimulate skeletal muscle build-up, while increasing fatty acid oxidation [54]. In this study, dietary supplementation of HMB markedly decreased the arterial concentration of TG. Notably, TG can be stored as lipid droplets within hepatocytes or secreted into the blood, but it also can be hydrolyzed, and the fatty acids may then be metabolized through the β-oxidation pathway in the liver [55]. The decreased arterial concentration of TG and numerically greater hepatic O_2_ consumption suggested that HMB supplementation increased fatty acid oxidation and the study revealed that this occurred in the muscles and in the liver, although the difference between CON and HMB sows could only be explained by the muscles. Similarly, Rittig et al. used indirect calorimetry to document that HMB administration induced a higher energy expenditure (heat production) and higher lipid oxidation rate [56]. It also increases lipolysis and decreases the content of adipose tissue with no change in body mass, leading to an increase of lipid availability. In addition, HMB (0.5 μM) combined with metformin and resveratrol markedly increases fat oxidation, sirtuin 1 activity, and adenosine monophosphate-activated protein kinase in muscle cells [57].

## 5. Conclusions

This study demonstrated that dietary HMB supplementation resulted in a high and fast net portal absorption of HMB, which in turn increased net portal uptake of amino acids and increased fatty acid oxidation through increasing plasma flow and insulin sensitivity during late gestation. Moreover, the data presented here suggest that 96% of the HMB ingested was metabolized/excreted/secreted in the body by a number of different organs, including GIT, liver, mammary gland and muscles. These data offer a dynamic metabolic track of orally administered HMB, which could provide essential guidance to improve HMB availability and efficacy to tissues for future research.

## Figures and Tables

**Table 1:** Feed and water intake of sows and performance of sows and piglets during transition and lactation, and apparent total tract digestibility of sow diets.

	CON	HMB	SEM	*p*-Values
Sows				
HMB intake from d –10 to farrowing, g/d	0.0	3.2	0.03	<0.001
Overall feed intake, kg/d	3.80	3.85	0.37	0.92
Water intake, L/d	26.0	24.8	6.9	0.89
Sow BW at d –10, kg	265	259	7.3	0.58
Sow BW loss from d –10 to 2, kg	20.3	15.0	2.9	0.23
Sow BW loss from d 2 to 28, kg	21.0	19.0	5.8	0.81
ATTD, %				
DM	83.5	82.2	1.0	0.28
GE	83.0	81.6	1.1	0.31
Nitrogen	84.2	83.5	1.3	0.63
Fat	62.7	58.4	2.2	0.13
Crude Fiber	68.2	65.9	2.4	0.45
Piglets				
Mean Piglet BW at birth, kg	1.24	1.20	0.04	0.45
Litter gain, kg/d	2.38	2.36	0.17	0.92
Litter size at birth	18.8	15.5	1.8	0.25
Litter size at d 2 (After cross-foster)	12.0	12.3	0.2	0.29
Litter size at d 28	11.7	11.0	0.7	0.56

CON = control; HMB = β-hydroxy-β-methyl butyrate; BW = body weight; ATTD = apparent total tract digestibility; DM = dry matter; GE = gross energy; SEM = standard error of the mean.

**Table 2:** Milk amino acids compositions of control (CON) and HMB-supplemented (HMB) sows during early and peak lactation.

Item	Treatment		SEM	DIM		SEM	*p*-Values		
	CON	HMB		3	17		Trt	DIM	Trt×DIM
Essential AA, g/kg milk									
Lysine	3.65	3.86	0.12	4.20	3.31	0.11	0.20	<0.001	0.80
Methionine	0.93	1.01	0.03	1.07	0.87	0.02	0.047	<0.001	0.75
Threonine	2.06	2.17	0.08	2.41	1.83	0.07	0.31	<0.001	0.71
Isoleucine	2.12	2.24	0.06	2.45	1.91	0.06	0.17	<0.001	0.72
Leucine	4.19	4.43	0.13	4.85	3.78	0.12	0.19	<0.001	0.70
Valine	2.74	2.89	0.09	3.18	2.45	0.09	0.24	<0.001	0.68
Histidine	1.33	1.41	0.04	1.54	1.20	0.04	0.17	<0.001	0.86
Phenylalanine	1.98	2.07	0.07	2.27	1.77	0.06	0.32	<0.001	0.62
Nonessential AA, g/kg milk									
Alanine	1.80	1.89	0.06	2.12	1.57	0.06	0.26	<0.001	0.60
Aspartate	4.11	4.39	0.12	4.77	3.73	0.12	0.12	<0.001	0.72
Cysteine	0.72	0.74	0.03	0.84	0.62	0.03	0.63	<0.001	0.31
Glutamate	9.80	10.55	0.28	11.00	9.36	0.26	0.07	0.002	0.99
Glycine	1.60	1.71	0.05	1.81	1.50	0.04	0.12	<0.001	0.94
Proline	5.32	5.78	0.17	5.92	5.18	0.16	0.06	0.008	0.90
Serine	2.68	2.88	0.10	3.10	2.47	0.09	0.16	<0.001	0.85
Arginine	2.37	2.52	0.08	2.76	2.14	0.08	0.18	<0.001	0.76

CON = control; HMB = β-hydroxy-β-methyl butyrate; Trt = treatment; DIM = days in milk; SEM = standard error of the mean; AA = amino acid.

**Table 3:** The arterial concentration of blood gases, hematocrit, and plasma metabolites of control (CON) and HMB-supplemented (HMB) sows during late gestation.

Item	Treatment		SEM	DIM		SEM	*p*-Values				
	CON	HMB		–10	–3		Trt	DIM	Trt×DIM	ST	Trt×ST
Whole blood											
Hematocrit, %	27.6	28.0	0.7	27.5	28.1	0.5	0.65	0.30	0.004	<0.001	0.04
O_2_, mmol/L	5.9	5.9	0.1	6.0	5.9	0.1	0.76	0.21	0.005	0.002	0.15
CO_2_, mmol/L	26.7	27.0	0.3	27.2	26.5	0.2	0.52	<0.001	0.24	<0.001	0.49
Plasma											
HMB, µmol/L	1.6	82.7	3.8	40.4	43.9	3.8	<0.001	0.53	0.47	<0.001	<0.001
Insulin, pmol/L	57.1	58.4	7.1	66.6	48.8	6.4	0.91	0.03	0.66	<0.001	<0.001
Glucose, mmol/L	5.4	5.2	0.1	5.2	5.3	0.1	0.28	0.24	0.13	<0.001	<0.001
Lactate, mmol/L	1.3	1.2	0.1	1.2	1.3	0.1	0.78	0.44	0.42	<0.001	0.002
Urea, mmol/L	4.0	3.7	0.4	3.8	3.9	0.3	0.64	0.47	0.21	<0.001	0.18
NEFA, µmol/L	167	114	21	121	160	19	0.13	0.08	0.86	0.006	0.71
TG, µmol/L	699	604	28	630	672	25	0.046	0.21	0.06	0.03	0.06
Essential AA, µmol/L											
Lysine	82	106	8.7	98	90	6.8	0.09	0.21	0.27	<0.001	0.003
Methionine	30	26	1.9	28	27	1.5	0.22	0.44	0.07	<0.001	0.17
Threonine	180	184	19.1	174	190	14.2	0.90	0.11	0.83	<0.001	0.008
Tryptophan	66	72	4.4	69	69	3.2	0.42	0.49	0.86	<0.001	0.009
Isoleucine	163	161	7.7	166	159	5.8	0.86	0.06	0.03	<0.001	0.006
Leucine	198	188	13.6	205	180	9.8	0.63	<0.001	0.01	<0.001	0.02
Valine	319	309	19.9	340	288	14.4	0.72	<0.001	0.06	<0.001	0.006
Histidine	86	78	5.5	82	82	4.0	0.32	0.70	0.71	<0.001	0.30
Phenylalanine	111	100	7.7	107	103	5.5	0.34	0.06	0.73	<0.001	0.003
Nonessential AA, µmol/L											
Alanine	573	570	48.6	563	581	35.8	0.97	0.40	0.71	<0.001	<0.001
Asparagine	25	20	2.4	24	21	1.8	0.26	0.009	0.02	<0.001	<0.001
Aspartate	41	41	2.4	42	40	1.8	0.91	0.20	0.88	<0.001	0.02
Cysteine	190	220	3.1	206	205	2.7	<0.001	0.68	0.17	0.001	0.98
Glutamate	240	249	11.2	254	235	9.3	0.56	0.08	0.40	<0.001	0.07
Glycine	917	886	39.8	875	928	32.6	0.60	0.15	0.71	<0.001	0.43
Proline	483	503	39.3	511	476	30.3	0.74	0.18	0.10	<0.001	0.14
Serine	151	138	9.2	148	142	6.7	0.35	0.08	0.02	<0.001	0.003
Tyrosine	119	116	8.4	121	114	6.1	0.82	0.03	0.86	<0.001	0.009
Glutamine	276	241	14.2	276	241	11.4	0.13	0.009	0.20	<0.001	0.04
VFA, µmol/L											
Acetate	400	376	18.0	401	375	14.0	0.38	0.04	0.83	<0.001	0.06
Propionate	2.5	3.7	0.8	4.6	1.6	0.8	0.28	0.008	0.94	0.54	0.65
Butyrate	6.7	7.1	1.1	6.5	7.4	0.8	0.82	0.15	0.92	0.34	0.05
Isobutyrate	9.4	8.4	1.1	9.4	8.3	0.8	0.55	0.08	0.37	0.006	0.39
Isovalerate	14.2	12.7	1.2	13.9	13.0	0.9	0.38	0.09	0.57	0.006	0.34

CON = control; HMB = β-hydroxy-β-methyl butyrate; Trt = treatment; DIM = days in milk; ST = sampling time; NEFA = non-esterified fatty acids; TG = triglycerides; VFA = volatile fatty acid; SEM = standard error of the mean; AA = amino acid.

**Table 4:** Blood and plasma flow, and net portal fluxes of gases and metabolites in control (CON) and HMB-supplemented (HMB) sows during late gestation.

Item	Treatment		SEM	DIM		SEM	*p*-Values				
	CON	HMB		–10	–3		Trt	DIM	Trt×DIM	ST	Trt×ST
Whole blood											
Portal flow, L/h	161	170	10	166	165	7	0.52	0.69	0.01	<0.001	0.03
O_2_, mmol/h	−441	−458	13	−457	−442	12	0.42	0.30	0.21	0.001	0.04
CO_2_, mmol/h	531	558	22	557	532	22	0.40	0.36	0.10	0.09	0.29
Plasma											
HMB, mmol/h	0.0	2.4	0.1	1.2	1.2	0.1	<0.001	0.80	0.73	<0.001	<0.001
Insulin, nmol/h	19	23	3.1	24	18	2.5	0.37	0.009	0.02	<0.001	<0.001
Glucose, mmol/h	267	309	21	286	290	18	0.29	0.93	0.50	<.0001	<0.001
Lactate, mmol/h	40	42	4.8	47	36	3.8	0.85	<0.001	0.21	<.0001	0.19
Urea, mmol/h	−5.6	−3.0	1.7	−3.5	−5.1	1.3	0.30	0.25	0.40	0.28	0.64
NEFA, mmol/h	−1.0	3.1	1.5	1.2	0.9	1.6	0.09	0.86	0.16	0.97	0.57
TG, mmol/h	−0.1	−3.5	2.7	−2.8	−0.8	2.8	0.44	0.64	0.85	0.51	0.60
Essential AA, mmol/h											
Lysine	5.2	5.9	0.3	5.5	5.5	0.3	0.20	0.82	0.12	<0.001	0.004
Methionine	1.2	1.1	0.2	1.2	1.1	0.1	0.69	0.42	0.003	<0.001	0.001
Threonine	3.9	4.4	0.3	4.1	4.2	0.3	0.35	0.98	0.40	<0.001	<0.001
Tryptophan	0.9	1.0	0.1	1.0	1.0	0.1	0.27	0.78	0.81	<0.001	0.05
Isoleucine	5.0	5.5	0.3	5.4	5.1	0.3	0.32	0.49	0.28	<0.001	<0.001
Leucine	8.8	9.6	0.4	9.3	9.0	0.4	0.30	0.44	0.20	<0.001	<0.001
Valine	6.6	7.2	0.5	6.8	7.0	0.4	0.49	0.76	0.35	<0.001	0.001
Histidine	2.3	2.3	0.2	2.4	2.2	0.2	0.85	0.57	0.27	<0.001	0.01
Phenylalanine	5.2	5.6	0.3	5.5	5.3	0.2	0.41	0.33	0.13	<0.001	<0.001
Nonessential AA, mmol/h											
Alanine	21.4	23.7	1.1	23.7	21.4	1.0	0.23	0.048	0.07	<0.001	<0.001
Asparagine	3.0	2.9	0.2	3.1	2.8	0.2	0.90	0.08	0.03	<0.001	<0.001
Aspartate	1.9	2.0	0.2	1.8	2.0	0.2	0.94	0.39	0.31	<0.001	0.48
Cysteine	2.5	3.0	0.1	2.7	2.8	0.1	0.005	0.83	0.75	<0.001	0.002
Glutamate	−1.5	−1.2	0.3	−1.5	−1.1	0.3	0.53	0.28	0.37	0.02	0.79
Glycine	18.0	19.3	1.4	19.2	18.1	1.2	0.54	0.31	0.19	0.002	0.74
Proline	11.2	12.7	0.9	12.3	11.7	0.8	0.27	0.37	0.08	<0.001	0.03
Serine	6.8	8.0	0.5	7.5	7.2	0.4	0.16	0.33	0.17	<0.001	<0.001
Tyrosine	3.3	3.6	0.2	3.5	3.4	0.2	0.37	0.44	0.19	<0.001	<0.001
Glutamine	3.3	3.8	0.9	3.5	3.6	0.8	0.69	1.00	0.06	<0.001	0.15
VFA, mmol/h											
Acetate	122.2	104.1	6.4	108.9	117.4	5.3	0.09	0.11	0.77	0.42	0.65
Propionate	56.6	47.6	5.9	52.4	51.8	4.5	0.33	0.76	0.10	0.67	0.38
Butyrate	21.9	17.0	2.0	18.2	20.7	1.6	0.12	0.08	0.53	0.33	0.46
Isobutyrate	1.6	1.6	0.1	1.7	1.5	0.1	0.74	0.26	0.52	0.86	0.19
Isovalerate	1.2	1.1	0.1	1.2	1.0	0.1	0.40	0.29	0.15	0.72	0.13

CON = control; HMB = β-hydroxy-β-methyl butyrate; Trt = treatment; DIM = days in milk; ST = sampling time; NEFA = non-esterified fatty acids; TG = triglycerides; VFA = volatile fatty acid; SEM = standard error of the mean; AA = amino acid.

**Table 5:** Net portal recovery of amino acids in control (CON) and HMB-supplemented (HMB) sows during late gestation.

Item	Treatment		SEM	DIM		SEM	*p*-Values		
	CON	HMB		–10	–3		Trt	DIM	Trt×DIM
Essential AA, %									
Lysine	64.8	75.4	4.2	70.5	69.8	4.2	0.10	0.91	0.18
Methionine	49.0	45.5	6.0	48.8	45.7	6.0	0.69	0.72	0.049
Threonine	54.3	61.5	4.6	57.4	58.4	4.6	0.28	0.88	0.47
Tryptophan	62.9	72.0	4.1	67.8	67.1	4.1	0.14	0.90	0.67
Isoleucine	61.9	68.5	3.5	66.8	63.6	3.5	0.21	0.53	0.29
Leucine	63.2	70.8	3.2	68.3	65.7	3.2	0.11	0.58	0.25
Valine	61.3	68.1	4.1	64.1	65.4	4.1	0.27	0.82	0.42
Histidine	57.9	58.7	4.7	62.0	54.6	4.7	0.92	0.28	0.26
Phenylalanine	67.8	73.7	3.5	72.1	69.3	3.5	0.26	0.59	0.23
Nonessential AA, %									
Alanine	181.4	210.9	9.0	207.6	184.7	9.0	0.04	0.10	0.23
Aspartate	15.5	19.8	0.8	17.7	17.6	0.8	0.003	0.97	0.96
Cysteine	51.6	53.0	5.8	49.6	55.1	5.8	0.87	0.52	0.63
Glutamate	−3.8	−3.4	0.5	−4.0	−3.3	0.5	0.64	0.40	0.57
Glycine	126.0	140.6	8.2	137.3	129.2	8.2	0.23	0.50	0.33
Proline	65.8	77.2	5.5	73.6	69.3	5.5	0.17	0.59	0.26
Serine	58.6	69.8	4.4	65.6	62.9	4.4	0.10	0.67	0.37
Tyrosine	86.0	95.2	4.9	92.2	89.1	4.9	0.21	0.66	0.25

CON = control; HMB = β-hydroxy-β-methyl butyrate. Trt = treatment; DIM = days in milk; SEM = standard error of the mean; AA = amino acid.

**Table 6:** Blood and plasma flow, and net hepatic fluxes of gases and metabolites in control (CON) and HMB-supplemented (HMB) sows during late gestation.

Item	Treatment		SEM	DIM		SEM	*p*-Values				
	CON	HMB		–10	–3		Trt	DIM	Trt×DIM	ST	Trt×ST
Whole blood											
O_2_, mmol/h	−487	−617	56.6	−567	−537	53.2	0.19	0.72	0.31	0.75	0.75
CO_2_^#^, mmol/h	397	546	84.0	514	428	68.0	0.33	0.26	0.16	0.52	0.53
RQ	0.83	0.90	0.05	0.90	0.84	0.05	0.47	0.65	0.57	0.06	0.53
Plasma											
Hepatic vein, L/h	197	260	12.0	226	232	12.1	0.002	0.76	0.43	0.49	0.92
Hepatic artery, L/h	38	90	15.7	60	68	14.8	0.06	0.70	0.87	1.00	1.00
HMB, mmol/h	0.00	−0.11	0.36	0.58	−0.69	0.35	0.44	0.03	0.02	0.24	0.26
Insulin, nmol/h	−15.3	−13.4	1.9	−15.3	−13.4	1.8	0.26	0.37	0.36	<0.001	<0.001
Glucose, mmol/h	19.3	7.9	15.6	25.7	1.5	15.2	0.76	0.28	0.46	0.08	0.79
Lactate, mmol/h	49.7	79.1	12.2	59.8	68.9	12.0	0.15	0.49	0.71	<0.001	0.26
Urea, mmol/h	45.9	38.6	3.9	45.3	39.3	3.0	0.23	0.006	0.17	<0.001	0.40
NEFA, mmol/h	−8.9	−3.1	2.4	−4.7	−7.3	2.2	0.11	0.41	0.86	0.59	0.23
TG, mmol/h	5.6	3.6	2.9	8.0	1.2	2.9	0.51	0.05	0.06	0.70	0.58
Essential AA, mmol/h											
Lysine	−1.0	−1.3	0.2	−1.0	−1.3	0.2	0.43	0.08	0.62	<0.001	0.44
Methionine	−0.1	0.6	0.1	0.3	0.2	0.1	<0.001	0.39	0.02	0.002	0.40
Threonine	−1.2	−0.9	0.2	−0.8	−1.2	0.2	0.17	0.18	0.30	0.12	0.04
Tryptophan	−0.6	−0.6	0.1	−0.5	−0.7	0.1	0.94	0.02	0.92	0.05	0.73
Isoleucine	−0.7	−0.8	0.3	−0.3	−1.1	0.3	0.80	0.047	0.88	0.006	0.46
Leucine	−1.0	−1.4	0.3	−1.0	−1.4	0.3	0.46	0.09	0.13	0.006	0.32
Valine	−0.7	0.1	0.5	0.4	−1.0	0.5	0.28	0.03	0.62	0.008	0.42
Histidine	−1.6	−1.5	0.3	−1.5	−1.5	0.3	0.60	0.93	0.28	0.84	0.84
Phenylalanine	−4.2	−3.7	0.2	−3.8	−4.1	0.2	0.06	0.83	0.73	<0.001	0.24
Nonessential AA, mmol/h											
Alanine	−25.4	−25.4	1.7	−25.7	−25.0	1.7	0.70	0.76	0.37	<0.001	0.03
Asparagine	−1.6	−1.3	0.1	−1.4	−1.5	0.1	0.04	0.45	0.77	<0.001	0.25
Aspartate	−0.9	−1.3	0.2	−1.2	−1.1	0.2	0.44	0.85	0.61	0.004	0.01
Cysteine	−2.0	−2.3	0.4	−1.9	−2.4	0.3	0.60	0.30	0.55	0.01	0.08
Glutamate	35.8	40.3	2.7	39.3	36.8	2.7	0.29	0.49	0.52	0.03	0.38
Glycine	−20.9	−14.7	2.0	−17.0	−18.5	1.7	0.07	0.33	0.84	0.85	0.54
Proline	−4.4	−4.2	1.1	−3.3	−5.3	1.0	0.92	0.11	0.38	0.19	0.68
Serine	−5.2	−5.6	0.7	−5.7	−5.1	0.6	0.99	0.34	0.25	<0.001	0.002
Tyrosine	−3.3	−2.8	0.3	−3.1	−3.1	0.2	0.21	0.92	0.57	<0.001	0.32
Glutamine	−1.6	−5.0	0.7	−3.3	−3.3	0.7	0.04	0.98	0.003	0.34	0.28
VFA, mmol/h											
Acetate	−8.3	−1.8	5.5	−8.9	−1.2	4.9	0.45	0.16	0.79	0.34	0.71
Propionate	−54.4	−51.2	7.2	−54.6	−51.1	5.4	0.67	0.29	0.27	0.43	0.33
Butyrate	−16.5	−15.1	2.9	−15.2	−16.4	2.2	0.68	0.37	0.78	0.31	0.76
Isobutyrate	−1.8	−1.6	0.2	−1.8	−1.6	0.2	0.43	0.55	0.90	0.39	0.28
Isovalerate	−1.2	−1.2	0.1	−1.3	−1.1	0.1	0.66	0.29	0.005	0.46	0.72

CON = control; HMB = β-hydroxy-β-methyl butyrate; Trt = treatment; DIM = days in milk; ST = sampling time; RQ = respiratory quotient; NEFA = non-esterified fatty acids; TG = triglycerides; VFA = volatile fatty acid; SEM = standard error of the mean; AA = amino acid. ^#^ Corrected for CO_2_ used for urea synthesis.

**Table 7:** Femoral extraction of metabolites of control (CON) and HMB-supplemented (HMB) sows during late gestation.

Item	Treatment		SEM	DIM		SEM	*p*-Values				
	CON	HMB		–10	–3		Trt	DIM	Trt×DIM	ST	Trt×ST
Plasma, %											
HMB	2.9	3.0	5.1	1.8	4.1	5.4	0.93	0.72	0.50	0.16	0.63
Insulin	14.4	4.3	3.6	12.3	6.3	3.1	0.09	0.08	0.92	0.02	0.47
Glucose	9.5	8.7	0.9	10.2	8.0	0.7	0.54	0.001	0.40	<0.001	0.004
Lactate	26.5	24.5	2.6	24.5	26.6	2.3	0.48	0.52	0.33	0.03	0.006
Urea	−0.8	−0.6	0.3	−0.6	−0.9	0.3	0.59	0.44	0.85	0.82	0.48
NEFA	13.0	−3.2	10.1	9.5	0.3	10.0	0.19	0.39	0.71	0.14	0.72
TG	0.0	1.6	2.5	0.8	0.9	2.3	0.57	0.82	0.27	0.83	0.38
Essential AA, %											
Lysine	13.5	5.8	3.4	12.2	7.1	2.5	0.17	<0.001	0.30	<0.001	0.001
Methionine	21.1	21.7	2.3	22.0	20.9	2.3	0.72	0.82	0.22	0.09	0.90
Threonine	3.8	1.8	1.0	3.4	2.1	0.8	0.20	0.13	0.55	0.006	0.08
Tryptophan	1.1	0.3	0.5	0.7	0.7	0.5	0.26	0.87	0.35	0.04	0.58
Isoleucine	5.3	2.5	1.4	4.6	3.3	1.1	0.21	0.19	0.39	0.001	0.01
Leucine	5.2	2.4	1.3	4.5	3.2	1.1	0.20	0.14	0.30	<0.001	0.10
Valine	3.2	2.0	0.9	3.2	2.1	0.7	0.43	0.16	0.97	0.02	0.04
Histidine	2.8	1.1	1.5	4.3	−0.4	1.5	0.51	0.04	0.41	0.53	0.68
Phenylalanine	2.9	1.3	1.2	2.6	1.6	1.0	0.48	0.23	0.39	<0.001	0.01
Nonessential AA, %											
Alanine	5.9	3.2	0.7	4.5	4.5	0.6	0.04	0.77	0.55	<0.001	0.02
Asparagine	10.8	5.8	2.2	8.8	7.7	2.1	0.18	0.68	0.77	0.005	0.23
Aspartate	10.4	6.5	2.9	8.6	8.3	2.3	0.38	0.79	0.65	0.04	0.33
Cysteine	−1.4	−0.8	0.4	−0.6	−1.6	0.4	0.23	0.10	0.83	0.07	0.02
Glutamate	27.1	17.3	3.4	23.8	20.5	2.5	0.09	0.03	0.89	0.92	0.51
Glycine	−0.0	−0.3	0.4	−0.2	−0.1	0.4	0.73	0.63	0.36	0.26	0.73
Proline	1.5	1.5	0.6	1.9	1.1	0.5	0.91	0.21	0.65	0.003	0.13
Serine	12.1	8.1	1.2	10.2	9.9	1.0	0.049	0.59	0.11	0.002	0.45
Tyrosine	1.9	1.0	1.1	1.9	1.0	0.9	0.57	0.19	0.18	0.01	0.26
Glutamine	−11.7	−12.4	1.7	−9.6	−14.5	1.7	0.78	0.04	0.87	0.35	0.37
VFA, %											
Acetate	59.9	53.6	4.7	54.0	59.4	3.7	0.36	0.07	0.96	0.63	0.47
Propionate	−830	242	410	281	−869	350	0.28	0.11	0.004	0.03	<0.001
Butyrate	9.0	9.4	12.1	8.7	9.7	12.1	0.85	0.81	0.33	0.09	0.57
Isobutyrate	72.4	60.4	4.9	68.4	64.3	4.2	0.11	0.27	0.26	0.15	0.64
Isovalerate	6.6	8.2	4.2	6.0	8.8	4.1	0.64	0.45	0.30	0.95	0.21

CON = control; HMB = β-hydroxy-β-methyl butyrate; Trt = treatment; DIM = days in milk; ST = sampling time; NEFA = non-esterified fatty acids; TG = triglycerides; VFA = volatile fatty acid; SEM = standard error of the mean; AA = amino acid.

## Supplementary Materials

The following are available online at https://www.mdpi.com/2072-6643/12/2/561/s1, Table S1: Dietary ingredients and chemical composition of the control diet.
