# Supplementary material for: Net Absorption and Metabolism of β-Hydroxy- β-Methyl Butyrate during Late Gestation in a Pig Model
Source: Nutrients. 2020 Feb 21;12(2):561. doi: 10.3390/nu12020561 (PMC7071374; doi:10.3390/nu12020561)
Supplement: Supplementary file 1 [file nutrients-12-00561-s001.pdf]

Supplementary Table 1. Dietary ingredients and chemical composition of the control diet

Ingredients, (as fed)	g/kg
Barley	40.00
Wheat	35.62
Soybean meal, toasted	18.09
Animal fat	3.00
Monocalcium phosphate	1.19
Calcium carbonate	1.55
Sodium chloride	0.36
Vitamin-mineral premix	0.20
Cromium oxide	2.27
Chemical composition	g/kg DM
Metabolizable energy	14.75 MJ/kg DM
DM, % (as fed)	91.18
Crude protein	193.64
Crude fat	55.44
Lysine	9.25 (7.87)
Methionine	2.88 (2.54)
Threonine	6.84 (5.64)
Tryptophan	2.35 (2.00)
Isoleucine	8.49 (7.28)
Leucine	14.39 (12.38)
Valine	9.95 (8.25)
Histidine	4.81 (4.16)
Phenylalanine	10.06 (8.73)
Alanine	8.13 (6.30)
Aspartate	16.45 (13.70)
Cysteine	3.59 (2.94)
Glutamate	44.11 (39.57)
Glycine	8.31 (6.57)
Proline	15.43 (11.96)
Serine	9.72 (8.23)
Tyrosine	5.58 (4.73)
Arginine	12.14 (10.90)

Composed to fulfill the recommendations for lactating sows by the Danish Pig Research Center (VSP, Copenhagen, Denmark).

[1]In parentheses: standardized ileal digestible (SID) content of individual AA (g/kg DM). The contents of SID Lys, Met, Thr, Ile, Leu, Val, His, Phe, and Cys were calculated based on feed ingredient compositions by DPRC (2013), and SID contents of Trp, Ala, Asp, Glu, Gly, Pro, Ser, Tyr and Arg were calculated from feed ingredient compositions by NRC (2012).
